# Using Interpolation to Estimate System Uncertainty in Gene Expression Experiments

**DOI:** 10.1371/journal.pone.0022071

**Published:** 2011-07-22

**Authors:** Lee J. Falin, Brett M. Tyler

**Affiliations:** 1 Virginia Bioinformatics Institute, Virginia Polytechnic and State University, Blacksburg, Virginia, United States of America; 2 Virginia Bioinformatics Institute and Department of Plant Pathology, Physiology, and Weed Science, Virginia Polytechnic and State University, Blacksburg, Virginia, United States of America; Inserm U869, France

## Abstract

The widespread use of high-throughput experimental assays designed to measure the entire complement of a cell's genes or gene products has led to vast stores of data that are extremely plentiful in terms of the number of items they can measure in a single sample, yet often sparse in the number of samples per experiment due to their high cost. This often leads to datasets where the number of treatment levels or time points sampled is limited, or where there are very small numbers of technical and/or biological replicates. Here we introduce a novel algorithm to quantify the uncertainty in the unmeasured intervals between biological measurements taken across a set of quantitative treatments. The algorithm provides a probabilistic distribution of possible gene expression values within unmeasured intervals, based on a plausible biological constraint. We show how quantification of this uncertainty can be used to guide researchers in further data collection by identifying which samples would likely add the most information to the system under study. Although the context for developing the algorithm was gene expression measurements taken over a time series, the approach can be readily applied to any set of quantitative systems biology measurements taken following quantitative (i.e. non-categorical) treatments. In principle, the method could also be applied to combinations of treatments, in which case it could greatly simplify the task of exploring the large combinatorial space of future possible measurements.

## Introduction

The widespread adoption in systems biology of high-throughput experimental assays designed to measure the entire complement of a cells genes or gene products in response to some set of experimental conditions has created a paradox. On one hand these techniques produce such large amounts of data that researchers often struggle to find meaningful and statistically significant patterns amongst the large amounts of noise. On the other hand, since the cost of producing one of these comprehensive measurements is relatively high, researchers are often limited as to the numbers of samples that they can afford to assay in their experimental design, even if the cost of collecting the sample material is relatively low. This may mean limiting the number of time points in a time course experiment, the number of different treatment levels, the number of biological or technical replicates, or all of the above. If the end goal is one of network inference or predictive model development, the scarcity of the measurements can lead to vastly under-determined systems.

In order to design the most useful possible experiment, a biologist needs information about the most dynamic regions of the system in response to each independent variable (e.g. time duration or treatment levels). Usually this is provided from the biologists domain knowledge and/or pilot experiments. However, a more efficient approach would be possible if the most dynamic or uncertain regions of the system could be predicted quantitatively. This would enable more measurements to be concentrated within the most dynamic or uncertain regions and fewer within the less dynamic or more certain regions. Uncertainty in a system can result from error or noise in those measurements that do exist, it can result from a lack of measurements in certain regions of the system, and it can result from intrinsic dynamics of the system in certain regions. Most statistical methods focus on estimating the uncertainty due to error in the existing measurements. There are also a number of methods to deal with uncertainty in model inference caused by noisy data, mostly by using either an automated or manual cyclic refinement of model parameters through parameter perturbation or “tuning” [1]–[3] or via Bayesian algorithms [4].

Many algorithms [5] have been developed to solve the problem where 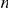 genes contain missing values at a certain time point. However, most of these approaches rely on the assumption that there exist 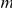 similar genes for which measurements are available at that same time point. For example, Bar-Joseph *et al.* [6] developed a method of estimating the value of unobserved data for a gene at a given time point by using continuous cubic B-spline representations of genes in the same class which contained observations for that time point.

Along with missing value imputation algorithms, algorithms for uncertainty quantification and minimization have been developed in a variety of fields. Lermusiaux [7] used the uncertainty estimates of the dominent processes in oceanic models to provide inputs to adaptive sampling algorithms. Singh *et al.* [8] used a similiar algorithm in systems biology by using the uncertainty in previously measured time points to choose the next time point to assay.

Another domain where this idea has been explored is that of robotic vision, where images of a 3D surface are taken and then certain regions of the surface are re-sampled at a higher resolution in order to decrease the level of uncertainty in those regions. Huang and Qian [9] used bicubic B-splines to represent the initial image of a 3D surface and then transformed the representation into a higher-dimensional B-spline surface using the uncertainty of the initial measurements as an added dimension. This new surface was then subdivided into smaller sections and the next best point for sampling identified using the geometric properties of each subsection.

Here, we describe a conceptually similar approach that may be used in conjunction with gene expression experiments, or other experiments where the cost of collecting samples is substantially less than the cost of assaying them. We introduce a novel algorithm to quantify the uncertainty in the unmeasured regions of gene expression time course experiments that is based on our (BTs) experience as a biologist regarding the dynamics of biological systems. The algorithm enables an experimental strategy where 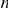 samples are collected but only 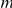 samples, 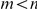, are initially assayed. Using the initial assay results, and plausible assumptions regarding the dynamics of the system, the algorithm creates a probability distribution that quantifies the uncertainty or potential dynamics of the system in the unassayed regions of the time course. Thus, for example, a researcher could use the information from this algorithm, to select additional unassayed samples for analysis that should add the most information to the system under study, iterating the process if necessary. To validate our approach, we use existing yeast gene expression datasets to show that our method provides accurate probabilistic predictions of gene expression values at unmeasured time points, in a model-independent way. While our approach was conceived in the context of time course measurements of gene expression profiles, the approach should be suitable for any set of biological measurements taken across any systematic set of quantitative treatments. The approach can also be applied to genome scale data sets through the use of dimensional reduction techniques such as Principal Components Analysis.

## Results

In any time course experiment, the region between measurements carries a certain degree of uncertainty. If a measurement is taken at some time point 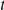 and another at time point 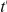, how the system behaves in the interval of time between these two time points is unknown. The first step in quantifying the uncertainty of the systems behavior in this unknown region is to calculate a comprehensive set of interpolations that represent the possible behavior of the system between these measurements.

**Figure 1:**
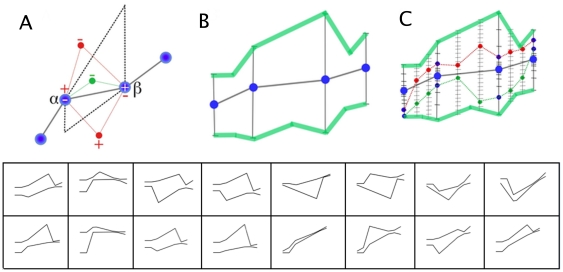
Establishing the likelihood bounds. (A) The plausible bounds (dashed in black) based on an arbitrary set of measured points (blue). “+” and “−” indicate the directions of changes of gradient. Red lines, subtended by red points, contain an additional inflexion point. Thin green line subtended by the green point does not. (B) Extended plausible bounds resulting from measurement error. The bounds (light green) consist of the union of 16 sets of boundaries (insets) defined by all possible combinations of the upper and lower confidence limits of the four measurements (blue dots). (C) After the extended plausible bounds have been determined, guide points (small blue dots) are inserted into each segment between the measured points. The span between plausible bounds for each guide point is divided into regular sub-intervals spanning the possible values (gray hatched lines). The confidence intervals for each measured point are divided into sub-intervals of equal probability (black hatched lines). To determine the likelihood distribution of plausible interpolations, one value is randomly chosen from each set of possible measured and guide points. If the interpolation passing through all these points contains more inflexion points than the original curve (e.g. the interpolation through the small red dots), it is discarded; otherwise (e.g. the interpolation through the small green dots) it is added to set of interpolations used to calculate the likelihood distribution.

After the boundaries containing the complete set of plausible interpolations have been established, the next piece of information required is the likelihood distribution of those interpolations within the boundaries. To obtain a useful approximation of the many possible interpolations within the boundaries, two randomly distributed “guide points” were considered to exist within each interval between measurement time points and between the upper and lower boundaries. New interpolations were defined that connected all measured points and guide points (Figure 1C), and were deemed plausible only if they contained no additional inflexion points. Biologically, the guide points could be thought of as potential new measurements.

Using this approach, the likelihood distribution of plausible interpolations at any time point could be calculated, using two methods. For clarity, the two methods are described in the following sections using the temporary assumption that the measured points have zero error. In the final two sections, a further elaboration to account for measurement error is introduced.

### Likelihood distribution of interpolations Monte Carlo method

While the complete set of interpolations could be infinite, our algorithm uses a simple, biologically plausible assumption to place reasonable bounds on the set of interpolations, namely that no novel regulatory event occurs within any interval unless there is experimental evidence for it. Mathematically we define this to mean that the total number of inflexion points in the actual path of the system through the measured points is assumed not to exceed the number of inflexion points implied by the original set of measurements. This assumption is an instance of Occams razor. (The limitations of this assumption are considered in the Discussion.).

Figure 1A shows the geometric boundaries that this constraint imposes on a small set of sample measurements. Here the measured points (blue points; assumed to have zero error) define the bounds of the constraint (dashed black lines). The line passing directly through the measured points in this example has two changes of gradient, a negative change at alpha followed by a positive change at beta, and thus a minimum of one inflexion point. If an interpolating curve passed through the small red point above the boundary, the gradient change at alpha would become positive, causing there to be two points of inflexion in the system. This would violate the constraint and the algorithm therefore assumes this curve to be implausible. Likewise, an interpolation that passes through a point below the boundary introduces a second inflexion point and is also assumed to be implausible. Defined in this way, the two boundaries therefore contain the set of all possible biologically plausible interpolations (as well as implausible ones with multiple inflexion points also). As an example of a plausible interpolation, the thin green line, passing through the green point in Figure 1A, does not contain more than one inflexion point.

### Biologically Plausible Interpolations

**Figure 2:**
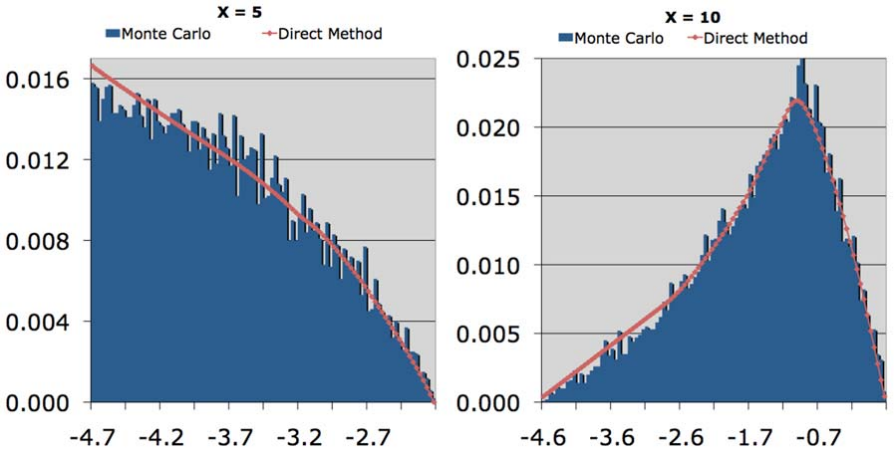
Estimation of the likelihood distribution. Estimation of the likelihood distribution via the Monte Carlo method compared to the Direct Method from a pilot study used in the development of the algorithm. The Monte Carlo method generated the blue approximation to the true distribution. The results of calculating the distributions directly using numerical integration are shown in red.

The first method used a Monte Carlo approach to calculate the likelihood distributions. In this method, the two guide points were spaced evenly along the time-axis (splitting each interval into exactly three sub-intervals) and randomly along the y-axis, constrained by the interpolation boundaries. The number of inflexion points of the interpolation passing through the real measurements and the guide points was then calculated. If the number of inflexion points of the new interpolation was greater than that of the original, the interpolation was discarded. If the interpolation was accepted, then the positions of all the guide points were recorded. This process was repeated until a predetermined number of passing interpolations had been found. The distributions of the accepted guide points in the y-direction at each point in the time axis thus approximated the distribution of all possible plausible interpolations passing through each time point. Because of the geometric relationships among neighboring guide points and measured points, the distributions of plausible interpolations were markedly non-uniform and non-normal (e.g. Figure 2).

This approach proved computationally expensive because the time required to discover sufficient passing interpolations to accurately calculate the likelihood distributions increased exponentially with the number of measured points.

### Likelihood distribution of interpolations Direct method

In order to create a computationally less expensive algorithm than the Monte Carlo method, we developed a method to calculate the likelihood distribution of interpolations directly from the geometric relationships among the measured points and guide points. The Direct method is based on exactly the same premises, but calculates the likelihood that plausible curves pass through a given location directly, rather than finding the likelihood by trial-and-error.

Given a set 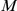 containing 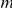 measured points (assuming initially they have no error), the algorithm first inserts 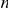 guide points in the intervals between the measured points (where 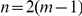) creating the set of guide points 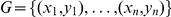 as for the Monte Carlo approach. The likelihood distribution of allowable guide points at 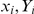, under the plausibility assumption, conditional on all other points may be determined by first calculating the likelihood distribution of 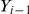 conditional on the set of distributions 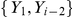 and the likelihood distribution of 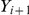 conditional on the set of distributions 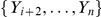. The likelihood distribution of 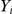 can then be calculated conditional on the distributions of 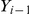 and 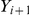 jointly. The likelihood distribution of 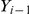 conditional on the preceding points may be calculated by the following method: At any position 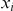, the likelihood distribution 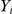 of any point in the y direction independent of the rest of the curve is assumed to be uniform. The likelihood distribution of 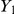 dependent on the preceding points is therefore uniform since there are no points preceding the point at 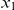 that may constrain the values of 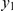. The distribution 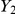, conditional on 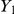 is calculated from the likelihood that a given 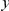 value at the next position (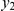 at 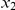) does not create a new inflexion point, given the measured points and the possible values of 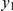, which can be derived from the (uniform) distribution 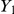 using the geometric relationships of each 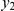 to each possible 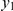 and the measured points that restrict where new guide points can be placed without creating additional inflexion points. By the same process, the likelihood that 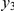 at 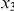 does not create a new inflexion point, given 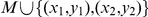, can be derived by numerical integration from the distribution of 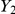 conditional on 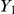 using the geometric relationships of each 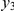 to each possible 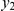. This procedure can be repeated until the distribution of 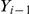 conditional on 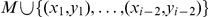 is obtained. The same procedure can be repeated, beginning with the uniform distribution of 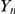, proceeding right to left until the distribution of 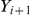 conditional on 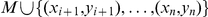 is obtained.

In order to compute the distributions via the method outlined above, using numerical integration, for each 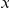 position, 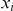, at which guide points will be inserted, the width of the interpolation envelope along the y-axis at that point was divided into 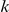 evenly spaced intervals (our implementation uses 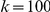), then each interval was represented by its midpoint, creating the set of midpoints 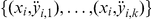.

Three probability values were defined for each interval, a probability conditional on the points to the right 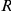, a probability conditional on the points to the left 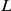, and a probability conditional jointly on the points to the left and right 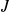. To begin the calculation, each of these values is set initially to 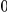, except for the sets of midpoints, 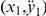 and 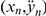 for which they are set to 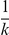.

To calculate the values for 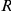, the algorithm first defines 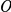 to be the interpolation passing through 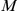. Then the algorithm processes the sets of possible guide points at each 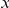 position in order right to left, starting with the guide points at 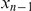, 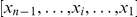 as described in the following pseudo code:

1. For every combination of midpoints 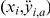 in 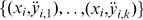 and 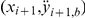 in 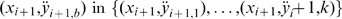:
  - Define 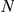 to be the interpolation passing through 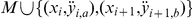.
  - If the total number of inflexion points in 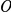 is the same as the total number of inflexion points in 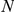, set 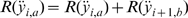.
2. Normalize 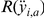 by setting 
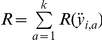
, and then dividing each 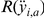 by 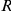 so the values now sum to 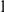.

The values for 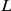 are calculated in a similar manner but are processed from right to left starting with the guide point at 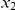, 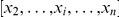 as follows:

1. For every combination of midpoints 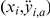 in 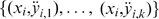
and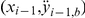 in 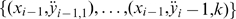:
  - Define 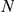 to be the interpolation passing through 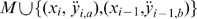.
  - If the total number of inflexion points in 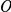 is the same as the total number of inflexion points in 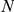, set 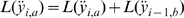.
2. Normalize 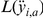 analogously to the normalization of 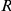.

Next, the values of 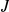 for the guide point intervals at 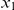 are set to be identical to the calculated 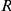 values and those for 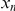 are set to be identical to the calculated 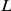 values.

Finally, the algorithm calculates the values for 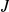 for the intervals at the remaining guide positions 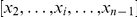 as follows:

1. For each combination of midpoints 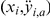 in 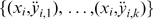, 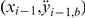 in 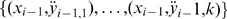, and 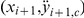 in 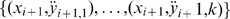:
  - Define 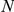 to be the interpolation passing through 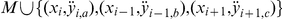.
  - If the total number of inflexion points in 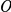 is the same as the total number of inflexion points in 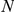, set 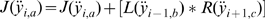.
2. Normalize 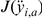 analogously to the normalization of 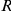.

### Accounting for measurement error in setting interpolation boundaries

As the algorithm uses the position of the measured points to define the boundaries of plausible interpolations, the error in these measurements must also be taken into account. To do so, the algorithm uses defined confidence limits of each measurement (we have used 99%) to define a plausible range of values for that point (as illustrated by the black vertical bars in Figure 1B). When there are 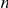 measured points, the upper and lower bounds of each of these 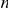 ranges are then used to calculate 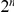 sets of possible boundaries (insets at bottom of Figure 1). The union of these sets is then taken as the boundaries for the set of plausible interpolations. (Light green bars in Figure 1B)

### Likelihood distribution of interpolations Accounting for measurement error

To take the measurement error into account in the calculation of the likelihood distributions, for each measurement 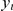 at 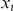, 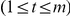 the algorithm uses the confidence interval for the measurement (our implementation uses 99%) as the range of plausible true values of the measurement 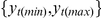, and divides the interval into 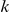 sub-intervals (we use 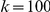) of variable width but equal probability, as follows. A probability value 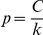 is assigned to each sub-interval, where 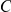 corresponds to the confidence level of the range 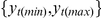. For 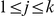, the algorithm calculates the width of each interval, 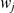 using the inverse t-distribution, 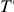, as follows:
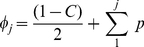
(1)


Then:
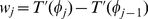
(2)(Figure 1C). In order to represent each interval by a point for the purposes of defined possible interpolations, the midpoint of each interval 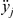 was defined as:
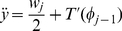
(3)


Since each measured point 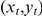 is now represented by a set of midpoints 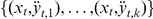, we modified the algorithm such that the calculation of the distribution of plausible guide points 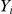, conditional on 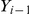 and 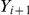 is calculated by systematically determining if guide points inserted at each of the points 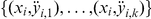 would create a new inflexion point in an interpolation spanning any set of points in the system of measured points and guide points (represented by interval mid-points), 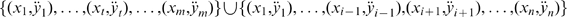.

In order to compensate for the added computational cost of this modification, which if implemented fully would increase the computational cost by a factor of 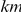, we employed a stratified sampling technique known as Latin Hypercube Sampling [10] to sample the set of all plausible true values of each measurement. It has been shown [11] that Latin Hypercube Sampling (LHS) requires only a small fraction of samples to be taken from a population in order to obtain an accurate estimate. LHS was implemented as follows:

For each set of midpoints spanning the confidence interval for a measurement 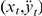, the algorithm permutes the midpoints of the 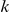 intervals using the Fisher-Yates shuffle [12]. Only the set of first ranked midpoints, the set of second-ranked midpoints, through to the set of 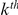-ranked midpoints (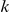 sets of measurement midpoints) are considered in the inflexion point analysis.

By employing Latin Hypercube Sampling, the added computational cost of the modification to account for measurement error is reduced to a factor of 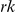, where 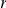 is the number of cycles of LHS used (our implementation uses 20 cycles).

### Evaluation

In order to evaluate the accuracy of the algorithm in predicting where new measurements may lie, including the calculated likelihood distributions, we looked for gene expression time course experiments with two qualities. First, we needed datasets that contained a relatively large number of measurements. This would allow us to provide the algorithm with only a fraction of the measurements and then measure how well the values of the omitted measurements corresponded to the likelihood distributions calculated by the algorithm. Second, in order to evaluate the effectiveness of the algorithm at accounting for measurement error, we needed a dataset that provided multiple replicates for each measurement so that confidence intervals could be assigned for each measurement.

### IRMA Dataset

Our first selected dataset comes from time series data recently published on the yeast IRMA gene network [13]. In addition to the previously mentioned criteria, the data generated by this network was also favorable because it comes from an in vivo gene network of known topology, which we could later use to apply our method to reverse engineering studies. In addition, some of the measurements displayed substantial amounts of variation, providing us with an opportunity to evaluate how substantial measurement error would affect the accuracy of the algorithms probabilistic prediction of gene expression values.

The authors [13] provided two sets of time series data, the “switch-on” data which consists of gene expression measurements obtained via RT-PCR when the cells were grown in the presence of glucose and then moved to a growth medium containing galactose, and the “switch-off” data which consists of expression measurements obtained when the cells were switched from a galactose medium to a glucose medium. Here we present the results of the analysis applied to the “switch-on” data. The results of the “switch-off” analysis can be found in the supplemental data (Data S1).

**Figure 3:**
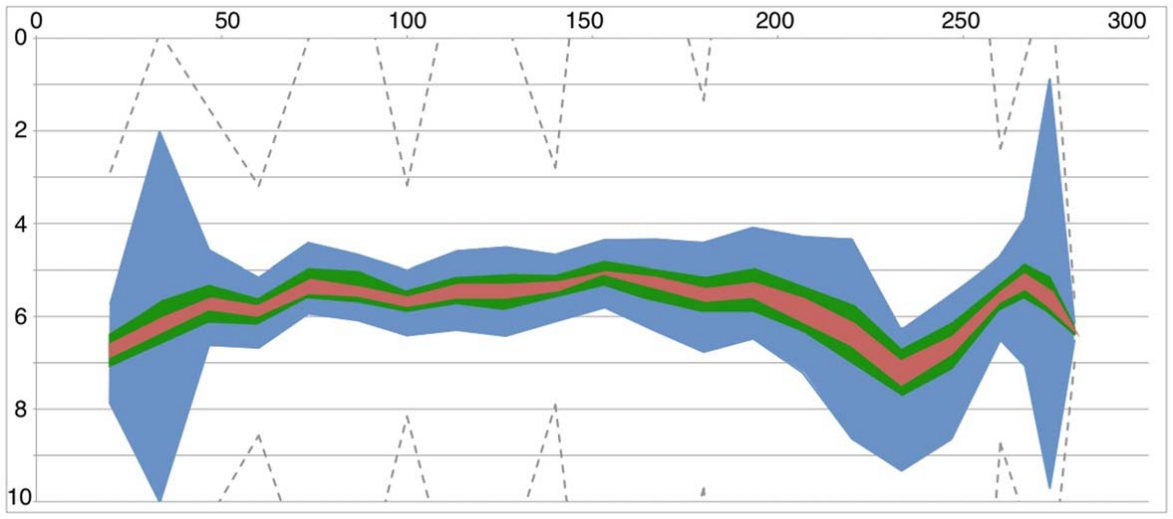
Likelihood distribution of biologically plausible interpolations for *ASH1*. The blue, green, and red regions indicate the 95%, 75%, and 20% likelihood regions respectively. The boundaries were created by using straight lines to connect the 20%, 75% and 95% likelihood boundaries of all the guide points and the 20%, 75% and 95% confidence boundaries of the measured points The gray dashed lines represent the interpolation boundaries as defined in Figure 1B using 99% confidence intervals of the measured points.

**Figure 4:**
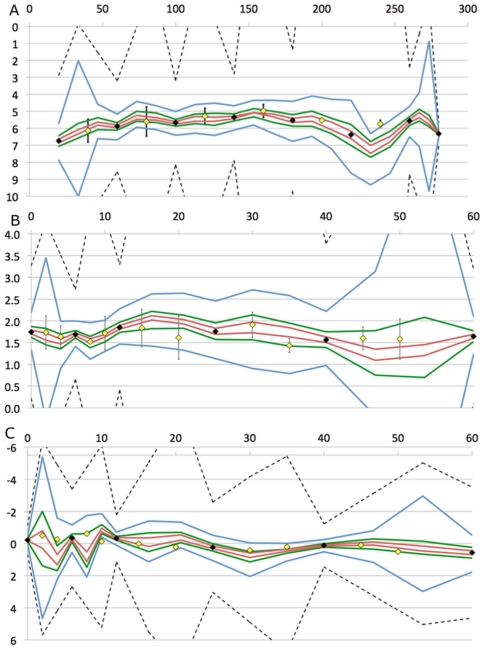
Prediction of omitted measurements. The blue, green, and red lines show the boundaries of the 95%, 75% and 20% likelihood regions respectively. The boundaries were calculated using only a fraction of the points (black diamonds). The omitted points (yellow diamonds) and their 95% confidence intervals are also shown. (A) *ASH1* expression in the IRMA Switch-On time series. (B) *YIL168W* expression in the NMD dataset. (C) The first principal component of the Extended NMD dataset.

We used RT-PCR data provided by the authors, which were obtained using the 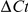 method. The authors averaged 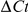 values across technical and biological replicates. Figure 3 shows the 20%, 75% and 95% likelihood boundaries for one of the five genes (*ASH1*) in the switch-on study. The graphs of the boundaries for the remaining genes can be found in the supplemental data (Data S2). In each case, every other time point was withheld from the dataset used to calculate the distribution of possible interpolations, and then the withheld measurements were compared to the predicted distributions. Figure 4A shows the likelihood regions for the *ASH1* gene that the algorithm calculated, along with the measured values of the omitted points (yellow diamonds). The 95% confidence intervals of the true locations of the measured points are also shown. The graphs for the remaining genes can be found in the supplemental data (Data S2). In the case of *ASH1*, 17% (1/6) of the measured points fell within the 20% likelihood envelope, and 83% (5/6) fell within the 75% and the 95% likelihood envelopes, even ignoring the error in the new measurements.

### Nonsense-Mediated Decay Dataset

Next we applied the algorithm to the time course expression data from a study [14] on nonsense-mediated mRNA decay. Our primary reason for choosing this study for analysis was the high quality of the microarray data provided, which used 3 biological replicates for each of the 16 time points.

The nonsense-mediated mRNA decay (NMD) pathway is responsible for the rapid decay of transcripts that contain a premature stop codon [15]. More recent studies have shown that the NMD pathway also helps to regulate the mRNA levels of a number of viable transcripts that do not contain premature stop codons [16].

We analyzed the time course expression data for 4 genes from a yeast strain containing a mutation in a gene that the authors identified as potential targets of NMD, *YIL164C*, *YIL165C*, *YIL167W*, and *YIL168W* [14] (GEO Series accession number GSE3076).

We generated expression values from the raw chip data by first correcting for optical noise using the GC-RMA [17] algorithm included in the Bioconductor package [18]. All pre-processing procedures were carried out using the open-source R software package [19].

Before generating the interpolation likelihood distributions we removed every 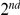 and 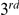 time point from the set of measurements, leaving only 6 of the original 16 measurements for the algorithm to use in construction of the likelihood distributions. We then plotted the locations of the omitted measurements against the likelihood distributions generated by the algorithm. The results for one of the four genes analyzed (*YIL168W*) are shown in Figure 4B. Plots of the other three genes may be found in the supplementary data (Data S3). Two of the ten points fell within the predicted 20% likelihood boundaries, and nine of the ten omitted points fell within the predicted 75% likelihood boundaries, even when the error of the omitted points was disregarded. All fell within the 95% likelihood boundaries, indicating that our interpolation-based method provided accurate predictions in this case also.

### Extended NMD Dataset

Finally, in order to test the algorithm on a high-throughput dataset, we applied the algorithm to the entire NMD mutant array series [14].

As before, expression values were generated from the raw chip data by first correcting for optical noise using the GC-RMA [17] algorithm included in the Bioconductor package [18]. Principal components analysis was then applied to the expression values of each replicate. The first principal component (accounting for 99% of the total variance) was selected for the analysis. All pre-processing procedures were carried out using the open-source R software package [19].

Before generating the interpolation likelihood distributions we removed every 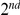 and 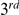 time point from the set of measurements, leaving only 6 of the original 16 measurements for the algorithm to use in construction of the likelihood distributions. We then plotted the locations of the omitted measurements against the likelihood distributions generated by the algorithm. The results are shown in Figure 4C. The data for the extended dataset can be found in the supplemental data (Data S4).

**Figure 5:**
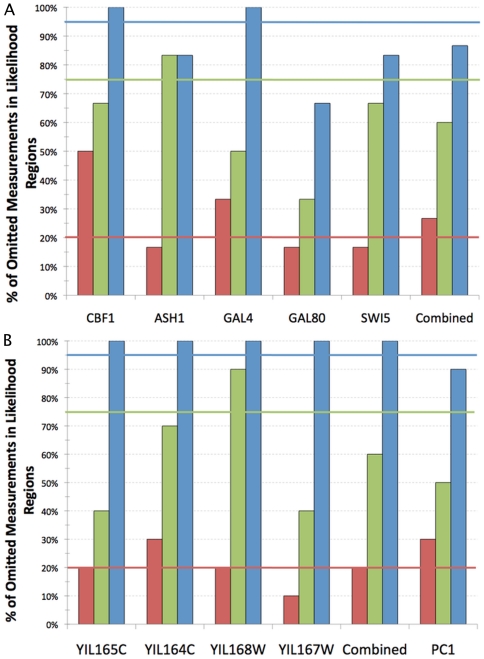
Accurate prediction of the locations of omitted measurements. The percentage of the omitted measurement means that were found within each likelihood region is shown for all measurements of each gene. The Combined group shows the average across all genes analyzed in the respective system. (A) The five genes from the switch-on dataset of the IRMA gene network. (B) The four genes selected from the NMD dataset and the first principal component of the Extended NMD dataset.

Figure 5 summarizes the overall accuracy of the algorithm in predicting future measurements for all three datasets. The frequency of successful predictions closely matched the estimated likelihood that future measurements would lie within a particular envelope, even ignoring the error inherent in the new measurements. In the IRMA dataset, 27% of predictions fell within the 20% likelihood envelope, 60% fell within the 75% likelihood envelope and 87% fell within the 95% likelihood envelope. Predictions for individual genes also followed this trend. For the four individual genes analyzed in the NMD dataset, the accuracy of the predictions was comparable: 20% of predictions fell within the 20% likelihood envelope, 60% fell within the 75% likelihood envelope and 100% fell within the 95% likelihood envelope. Finally the results for the first principal component of the extended NMD dataset shows that 30% of the predictions fell within the 20% likelihood envelope, half fell within the 75% likelihood envelope, and 90% fell within the 95% likelihood envelope.

## Discussion

The ability to quantify which gaps in a dataset contribute the most to the uncertainty in the knowledge about a particular system provides a powerful tool for planning future experimentation, especially when the cost of the future experiments is high. By utilizing a simple but powerful assumption about the plausible behaviour of biological systems, we have created an algorithm that quantifies the uncertainty created by gaps in biological datasets in a probabilistic fashion, including an intuitive graphical representation (illustrated in Figure 3). The following is an example of how this approach could be utilized in practice. If a biologist has collected tissue samples at 50 time points (ideally with three or more replicates), but only has a budget for assaying mRNA levels at 20 of those time points, mRNA levels from an initial set of 5 to 10 time points, analyzed using this algorithm, could provide the researcher with guidance as to which additional time points should be assayed to provide the most useful additional information about the system. In principle, the biologist could add the additional time points one by one, recomputing the predictions each time, or else add larger numbers of additional measurements at each iteration. This particular use of the algorithm avoids additional uncertainties introduced by repeating the entire experiment, with replicates, each time new measurements are needed.

We have presented two possible uses of our algorithm. First, we have shown its use to estimate the likelihood distributions of new measurements of the levels of individual genes. Second by using a summarization method (here we used Principal Components Analysis), we have shown its use in estimating the likelihood distribution of interpolations for a genome-wide microarray dataset. Several approaches can be used to estimate the uncertainty of the whole system at each time point. For example, the range of a particular quantile (e.g. 95%) for each gene at each time point could be used, or if it was considered desirable to take the size of the gap between measured time points into account, the area of the likelihood envelope between two measured points could be used. The range (or envelope area) of a particular gene of interest could be used to target a new measurement, or the ranges for all (or selected) genes in the system could be combined (e.g. by multiplication) to calculate an aggregate uncertainty. If time points were being considered that fell between measured points or guide points, then a simple interpolation (e.g. linear or cubic spline) could be used to estimate the distribution at the point of interest. Unlike sensitivity analysis techniques that attempt to measure the sensitivity of models to changes in parameter values or initial conditions [2], our algorithm does not require a model, since it analyzes the uncertainty based solely on the input data, in a model-agnostic way. On the other hand, if a model is available, and the parameters can be rapidly and automatically estimated from the data, our approach could be combined with model-building algorithms to create uncertainty distributions in the inferred model parameters. Our algorithm represents an instance of the general approach described by Hutter [20]. By employing an assumption that plausible interpolations through the data have no additional inflexion points, and by using pairs of guide points as a device to discover a representative set of plausible interpolations, we have rendered the space of all possible interpolations computable and quantifiable. A related approach has been used for robotic vision [9], however our approach has the advantage of also calculating the likelihood distributions of the system within unknown regions, as illustrated in Figure 4.

One limitation of our algorithm is the assumption that in order for an interpolation to be “biologically plausible” it must not introduce new, unmeasured regulatory events into the system, which is an instance of Occams razor. In cases where the initial measurements have been spaced far apart, there is obviously an increased likelihood that this assumption might be invalid, and biologists should take this into account in planning future experiments.

For biologists interested in “take-home messages” from this study, two are immediately evident: (i) since the predictions of the system are affected by the reliability of the measurements, the more replicates of the measured points the better (good advice in any context); (ii) intervals that contain an inflexion point are usually the most uncertain, and thus the best place for new measurements, because of the additional freedom in plausible paths this allows.

Although the context for developing the algorithm was gene expression measurements taken over a time series, the approach can be readily applied to any set of quantitative systems biology measurements taken following quantitative (i.e. non-categorical) treatments. In principle, the method could also be applied to combinations of treatments, in which case it could greatly simplify the task of exploring the large combinatorial space of future possible measurements. This methodology should have wide applications outside of biology as well. Our approach can benefit any application that uses continuous sets of measurements (e.g. time course studies), where the system under question can be expected to be constrained in a predictable fashion, and where it is desirable to quantify the uncertainty in the intervals between measurements.

## Methods

The Castor analysis software developed to calculate the likelihood distributions is available under the GNU GPL from: http://vmd.vbi.vt.edu/download/software/index.php System Requirements: Mac OS X 10.5 or higher.

## Supporting Information

Data S1 **IRMA Switch-Off Data.** Results of the analysis for all five genes of the IRMA “Switch-Off” dataset.
(XLSX)

Data S2 **IRMA Switch-On Data.** Results of the analysis for all five genes of the IRMA “Switch-On” dataset.
(XLSX)

Data S3 **Nonsense-Mediated Decay Data.** Results of the analysis for the Nonsense-Mediated Decay dataset.
(XLSX)

Data S4 **Extended Nonsense-Mediated Decay Data.** Results of the analysis for the Extended Nonsense-Mediated Decay dataset.
(XLSX)

## References

[1] Challinor A, Wheeler T (2005). Quantification of physical and biological uncertainty in the simulation of the yield of a tropical crop using present-day and doubled co2 climates.. Philosophical Transactions of the Royal Society B.

[2] Oberkampf WL, DeLand SM, Rutherford BM, Diegert KV, Alvin KF (2002). Error and uncertainty in modeling and simulation.. Reliability Engineering & System Safety.

[3] Palmer T (2000). Predicting uncertainty in forecasts of weather and climate.. Reports on Progress in Physics.

[4] Christie M, Demyanov V, Erbas D (2006). Uncertainty quantification for porous media flows.. Journal of Computational Physics.

[5] Brock GN, Shaffer JR, Blakesley RE, Lotz MJ, Tseng GC (2008). Which missing value imputation method to use in expression profiles: a comparative study and two selection schemes.. BMC Bioinformatics.

[6] Bar-Joseph Z, Gerber GK, Gifford DK, Jaakkola TS, Simon I (2003). Continuous representations of time-series gene expression data.. Journal of Computational Biology.

[7] Lermusiaux PF (2006). Uncertainty estimation and prediction for interdisciplinary ocean dynamics.. Journal of Computational Physics.

[8] Singh R, Palmer N, Gifford D, Berger B (2005). Active learning for sampling in timeseries experiments with application to gene expression analysis.. http://portal.acm.org/citation.cfm?id=1102351.1102456.

[9] Huang Y, Qian X (2008). An efficient sensing localization algorithm for free-form surface digitization.. Journal of Computing and Information Science in Engineering.

[10] McKay M, Beckman R, Conover W (1979). A comparison of three methods for selecting values of input variables in the analysis of output from a computer code.. Technometrics.

[11] Yu P, Yang T, Chen S (2001). Comparison of uncertainty analysis methods for a distributed rainfall–runoff model.. Journal of Hydrology.

[12] Durstenfeld R (1964). Algorithm 235: Random permutation.. Communications of the ACM.

[13] Cantone I, Marucci L, Iorio F, Ricci MA, Belcastro V (2009). A yeast synthetic network for in vivo assessment of reverse-engineering and modeling approaches.. Cell.

[14] Guan Y, Myers CL, Hess DC, Barutcuoglu Z, Caudy AA (2008). Predicting gene function in a hierarchical context with an ensemble of classifiers.. Genome biology.

[15] Pulak R, Anderson P (1993). mrna surveillance by the caenorhabditis elegans smg genes.. Genes & development.

[16] Culbertson M, Leeds P (2003). Looking at mrna decay pathways through the window of molecular evolution.. Current opinion in genetics & development.

[17] Wu Z, Irizarry R, Gentleman R, Murillo FM, Spencer F (2004). A model-based background adjustment for oligonucleotide expression arrays.. Journal of the American Statistical Association.

[18] Gentleman RC, Carey VJ, Bates DM, Bolstad B, Dettling M (2004). Bioconductor: open software development for computational biology and bioinformatics.. Genome biology.

[19] Team R (2008). R: A language and environment for statistical computing.. http://www.R-project.org.

[20] Hutter M (2005). Universal Artificial Intelligence: Sequential Decisions Based On Algorithmic Probability..

